# Fluorescence Lymphography Using Indocyanine Green During Esophagectomy for Cancer to Prevent Chyle Leakage: A Propensity Score Matched Analysis

**DOI:** 10.1245/s10434-026-19359-w

**Published:** 2026-03-19

**Authors:** Sofie P. G. Henckens, Dillen C. van der Aa, David J. Nijssen, Freek Daams, Wietse J. Eshuis, Mark I. van Berge Henegouwen, Suzanne S. Gisbertz

**Affiliations:** 1 Department of Surgery, Amsterdam UMC, Location University of Amsterdam, Amsterdam, The Netherlands; 2 Cancer Center Amsterdam, Cancer Treatment and Quality of Life, Amsterdam, The Netherlands; 3 Department of Gastroenterology and Hepatology, Amsterdam Gastroenterology Endocrinology Metabolism, Amsterdam UMC, Location University of Amsterdam, Amsterdam, The Netherlands; 4 Department of Surgery, Amsterdam UMC, Location Vrije Universiteit, Amsterdam, The Netherlands

**Keywords:** Esophagectomy, Minimally invasive surgery, Thoracic duct, Indocyanine green, Chyle leakage

## Abstract

**Background:**

This study evaluated the efficacy of intraoperative fluorescent lymphography with indocyanine green (ICG) to reduce the incidence of chyle leakage post-esophagectomy.

**Methods:**

This prospective observational cohort study was conducted among patients who underwent fluorescence lymphography during esophagectomy for cancer between May 2022 and August 2023 at a single tertiary referral center. After 1:3 propensity score matching, the results were compared between 59 patients who underwent fluorescence lymphography (ICG group) and a historical cohort who did not (non-ICG group). The primary outcome was the incidence of postoperative chyle leakage.

**Results:**

The study included 59 patients in the ICG group and 177 non-ICG controls. ICG was ultrasound guided bilaterally injected into inguinal lymph nodes in 26 patients (44%), the small bowel mesentery in 30 patients (51%), and both sites in three patients (5%). Thoracic duct visualization was successful in 85%. Fluorescence lymphography influenced intraoperative management in 21 patients (36%), with placement of additional clips. The incidence of chyle leakage was 17% (10/59) in the ICG group and 10% (18/177) in the non-ICG group (*p* = 0.163). All patients with chyle leakage in the ICG group were treated conservatively versus two re-interventions in the non-ICG group (*p*=0.271).

**Conclusions:**

Real-time ICG fluorescence lymphography is a promising tool for the intraoperative detection and management of chyle leakage during esophagectomy, although no reduction in chyle leakage was demonstrated. Further studies are required to elucidate the efficacy of fluorescence lymphography with ICG in reducing the incidence of postoperative chyle leakage.

**Supplementary Information:**

The online version contains supplementary material available at 10.1245/s10434-026-19359-w.

Esophagectomy with or without neoadjuvant chemo(radio)therapy is the standard curative treatment for esophageal cancer and frequently incorporates en-bloc thoracic duct resection for oncological purposes. Thoracic duct resection facilitates complete mediastinal lymphadenectomy and negative margins.^1^ Furthermore, ligation and resection of the duct is considered a preventive measure against postoperative chyle leakage.^2^ The thoracic duct transports chyle from below the diaphragm back into the circulatory system via the subclavian vein.^3^ Its intricate anatomy, along with the presence of collaterals and side branches, poses significant challenges for accurate intraoperative visualization and makes ligation difficult.^4^

Chyle leakage is a common complication after esophagectomy, occurring in 2–21% of cases.^5^ It arises from intraoperative damage to the thoracic duct or its tributaries, resulting in postoperative persistent lymphatic fluid leakage into the pleural or abdominal spaces.^6–9^ This condition can lead to prolonged thoracic drainage, extended hospital stay, additional re-interventions, dietary restrictions, nutritional deficits, increased healthcare costs, diminished quality of life, and even decreased long-term survival.^5,10^ In addition to the wide variation in reported incidence rates of chyle leakage, literature also highlights considerable variability in the proportion of patients requiring surgical intervention for this condition – either as primary treatment or following failure of conservative management – with percentages reported to be as high as 41%.^5,11,12^

Traditional management of chyle leakage is predominantly reactive rather than preventive. Integration of fluorescence lymphography using indocyanine green (ICG) during esophagectomy offers a potential preventive strategy by enabling accurate visualization of the thoracic duct and its collaterals. Consequently, this technique might facilitate intraoperative identification of chyle leakage and allow for immediate surgical intervention to address leakage, potentially reducing the postoperative incidence.^13^ At the initiation of this study, some small series carefully indicated that ICG fluorescence lymphography is feasible for visualizing the thoracic duct and thereby possibly reducing the incidence of chyle leakage.^14^ However, robust large comparative studies were lacking. The objective of this study was to assess the efficacy of fluorescence lymphography with ICG in preventing postoperative chyle leakage after minimally invasive esophagectomy. It was hypothesized that this approach would lead to a reduction in postoperative incidence of chyle leakage.

## Methods

### Study Design and Patient Population

This prospective observational cohort study, conducted at a tertiary referral center, utilized propensity matching analysis to compare outcomes between two groups undergoing esophagectomy: the non-ICG group (January 2013 to November 2023) and the ICG group (May 2022 to August 2023). Eligible patients were aged >18 years and underwent total minimally invasive or robot-assisted minimally invasive Ivor Lewis or McKeown esophagectomy for resectable esophageal carcinoma (cT1-4a, N0-3, M0). Exclusion criteria included allergy to ICG, iodide, or sodium iodide; other contraindications for ICG (e.g. hyperthyroidism, recent thyroid radioactive iodide exposure); and history of inguinal lymphadenectomy. The study was conducted according to the principles of the Declaration of Helsinki and in accordance with the Medical Research Involving Human Subjects Act, and written informed consent was obtained. Results were reported following the Strengthening the Reporting of OBservational studies in Epidemiology statement guidelines.^15^

### ICG Administration and Fluorescence Lymphography Protocol

A 25 mg vial of ICG powder (Diagnostic Green, VERDYE) was dissolved in 10 ml of sterile water, obtaining a solution of 2.5 mg/ml. ICG administration varied by esophagectomy type. During Ivor Lewis procedures, ICG was injected in the abdominal phase (in the root of the small bowel mesentery) or post-abdominal phase (bilateral inguinal lymph node injection). During McKeown procedures, ICG was injected after induction of anesthesia (bilateral inguinal lymph node injection).

A bolus of 2 ml reconstituted ICG was injected bilaterally (10 mg total per patient) into inguinal lymph nodes under ultrasound guidance. Next, initially during the study, 50 ml of enteral cream was administered via the feeding jejunostomy (created during the abdominal phase, in case of Ivor Lewis esophagectomy) to stimulate chyle flow, as fasting suppresses lymphatic activity. However, this practice was discontinued after the first 20 cases (33.8%), because, including in McKeown esophagectomy wherein administration of enteral cream was impossible, accurate visualization of the thoracic duct was achieved and so was found to have no added value. In case of ICG injection in the mesentery, 2 ml of reconstituted ICG was injected using a 25-gauge needle into the root of the small bowel mesentery just below the peritoneum. Intraoperative imaging of fluorescence signal was performed with the Stryker 1588 AIM, Near InfraRed (NIR) camera or Firefly NIR camera, da Vinci Xi, Intuitive.

During the thoracic phase, the thoracic duct was routinely double clipped 2–3 cm above the diaphragm and at the level of the arch of the azygos vein using metal clips (conventional minimally invasive esophagectomy) or hemolocks (robot-assisted esophagectomy) and transected under fluorescence guidance. The operating area was then inspected for chyle leakage with fluorescence lymphography directly after thoracic duct ligation and just before terminating the procedure. Any detected leakage was immediately treated with additional clipping or suture ligation. At the end of the thoracic phase, a Jackson–Pratt drain and a chest tube were placed in the right pleural cavity for postoperative drainage.

### Lymphadenectomy

In our center, lymphadenectomy during transthoracic esophagectomy is performed according to a standardized protocol. In the abdominal phase (either laparoscopic or robotic-assisted), this includes upper abdominal lymph node dissection (station 14–19 according to the TIGER classification).^16^ For intrathoracic anastomosis, the standardized procedure consists of mediastinal lymph node dissection (station 8, 9, 11, 12 and 13). In case of a cervical anastomosis, the procedure began with the thoracic phase, comprising mediastinal lymphadenectomy (station 4, 5, 8, 9, 10, 11, 12 and 13). When indicated, a cervical lymph node dissection was performed (station 2). The abdominal phase was then performed as described above. During the inclusion years of the historical cohort, the technique and extent of standard lymphadenectomy changed over time.^17^ In 2013, standard dissection of right paratracheal (4R) and aorto-pulmonary window^5^ lymph nodes and backtable dissection and separate lymph node submission to pathology was initiated. In 2013, a minimum lymph node yield of 15 became a quality indicator in the Dutch Upper Gastrointestinal Cancer Audit (DUCA). In 2014, standard lymphadenectomy was extended with left paratracheal (4L) lymph nodes. As a result, the lymph node yield showed some variation between 2013 and 2023.

### Postoperative Care

All patients received a surgically placed jejunostomy tube to secure early postoperative enteral feeding according to a standardized protocol. On postoperative days 0 and 1, patients were kept nil by mouth, with nasogastric tube in situ. On postoperative day 1, tube feeding through jejunostomy was started and increased over the next few days according to the dietitian’s plan. On postoperative day 3, an upper gastrointestinal contrast passage evaluation was performed. In case of passage through the gastric conduit, the nasogastric tube was removed and oral intake was initiated with clear liquids (International Dysphagia diet Standardisation Initiative [IDDSI] level 1, up to 150 ml every 2 h for the first 24 h), followed by a smooth liquid diet (IDDSI levels 0–4, maximum 150 ml every 2 h).^18^ For the first 2 weeks postoperatively, until outpatient follow-up, patients adhered to a liquid and pureed diet (IDDSI 0–4), increasing to maximum 250 ml every 2 h. In addition, nocturnal tube feeding via jejunostomy was continued at home at least until the first outpatient clinic visit.

Drain production and fluid characteristics were monitored daily. The thoracic drain was removed on day 1 if there was no evidence of air leakage and lung expansion was confirmed on chest X-ray. The Jackson–Pratt drain was removed when output was below 200 ml/24h. If chylous leakage was suspected (based on milky aspect or volume >400 ml/24h from day 4 onwards), the drain fluid was analyzed for triglycerides.

### Follow-up

All patients were intensively monitored during hospital admission. The occurrence of postoperative complications, including chyle leakage, and outcomes were recorded until the first outpatient visit at 2 weeks after discharge, in line with national guidelines.

### Definitions and Outcomes

Chyle leakage was defined according to the Esophagectomy Complications Consensus Group (ECCG) criteria as triglycerides ≥1.13 mmol/L in the drain fluid and/or each milky aspect of drain fluid for which a medium chain triglyceride diet or total parenteral nutrition was initiated.^19^ Chyle leakage was classified as type I when managed with enteric dietary modifications, type II when total parenteral nutrition was required, and type III when interventional or surgical therapy was necessary. Severity was categorized as level A for output <1L/24h and level B for output >1L/24h.^7^

Primary outcomes were the incidence and characteristics of postoperative chyle leakage. Secondary outcomes included change of operative management, hospital stay, readmission rate, (positive) lymph node yield, and radicality.

### Data Collection and Statistical Analyses

Baseline demographics, operative details, and short-term postoperative outcomes were prospectively recorded and analyzed. The electronic case report form (eCRF) collected data on ICG administration site, quality of thoracic duct visualization, change of management, duration of thoracic drainage, and adverse events. Propensity score matching was used to minimize confounding between the ICG and non-ICG groups. A logistic regression model based on baseline characteristics (Table 1) generated the propensity score. Analyses were followed by one-to-three nearest-neighbor matching without replacement using a caliper of 0.25 multiplied by the standard deviation of the logit to weed out bad matches. The mean standardized differences were used to evaluate the covariate balance of the matched cohort; discrepancies <10% were considered to indicate good balance. Categorical data were reported as counts and percentages, continuous data were shown as either mean ± standard deviation (or range) or as median and range, depending on distribution of the data. Categorical and continuous variables were compared using the chi-squared test and the Mann–Whitney U test, respectively. Correlations were assessed using Pearson’s or Spearman’s coefficient based on data distribution. Statistical analyses were conducted using SPSS (IBM Statistics, version 28.0). A *p*-value ≤0.05 was considered statistically significant.

**Table 1:** Characteristics of the study population before and after matching

Characteristics	Before matching			After matching			SMD
	ICG lymphography (*N* = 60)	Non-ICG lymphography (*N* = 795)	*p*-Value	ICG lymphography (*N* = 59)	Non-ICG lymphography (*N* = 177)	*p*-Value	
Male sex	49 (81.7)	618 (77.7)	0.478	48 (81.4)	140 (79.1)	0.709	0.05
Age, years	66.6 ± 9.1	64.9 ± 8.8	0.160	67.0 ± 8.4	66.7 ± 9.1	0.827	0.03
BMI, kg/m^2^	24.9 ± 3.9	25.7 ± 4.3	0.194	25.8 ± 4.7	24.8 ± 3.7	0.118	–
ASA							
I	0 (0)	156 (19.6)	**0.003**	0 (0)	0 (0)	0.751	0.05
II	41 (68.3)	456 (57.3)		40 (67.3)	116 (65.5)		
III	19 (31.7)	174 (21.9)		19 (32.2)	61 (34.5)		
IV	0 (0)	2 (0.3)		0 (0)	0 (0)		
Missing	*0 (0)*	*8 (1.0)*		*0 (0)*	*0 (0)*		
cT stage							
T0	0 (0)	0 (0)	0.780	0 (0)	0 (0)	0.609	–
T1	4 (6.7)	43 (5.6)		4 (6.8)	9 (5.1)		
T2	9 (15.0)	157 (20.4)		9 (15.3)	33 (18.8)		
T3	46 (76.7)	544 (70.6)		46 (78.0)	128 (72.7)		
T4	1 (1.7)	11 (1.4)		0 (0)	5 (2.8)		
Missing	*0 (0)*	*11 (1.4)*		*0 (0)*	*1 (0.6)*		
cN stage							
N0	20 (33.3)	298 (38.1)	0.532	20 (33.9)	65 (36.7)	0.689	0.06
N1	28 (46.7)	296 (37.9)		28 (47.5)	76 (42.9)		
N2	10 (16.7)	161 (20.6)		9 (15.3)	33 (16.7)		
N3	2 (3.3)	12(1.5)		2 (3.4)	2 (1.1)		
Missing	*0 (0.0)*	*15 (2.0)*		*0 (0.0)*	*1 (0.6)*		
Tumor localization							
Proximal	3 (5.0)	11 (1.4)	**0.001**	3 (5.1)	2 (1.1)	**0.007**	–
Mid	1 (1.7)	115 (14.4)		1 (1.7)	17 (9.6)		
Distal	52 (86.7)	547 (68.7)		51 (86.4)	126 (71.2)		
GEJ	4 (6.7)	123 (15.5)		4 (6.8)	32 (18.1)		
Preoperative histology							
Adenocarcinoma	56 (93.3)	610 (77.1)	**0.012**	55 (93.2)	142 (80.2)	0.058	–
SCC	3 (5.0)	160 (20.2)		3 (5.1)	31 (17.5)		
Other	1 (1.7)	21 (2.7)		1 (1.7)	4 (2.3)		
Neoadjuvant chemoradiotherapy							
Yes	56 (93.3)	684 (85.9)	0.256	55 (93.2)	166 (93.8)	0.878	0.02
No	4 (6.7)	106 (13.3)		4 (6.8)	11 (6.2)		
Missing	*0 (0)*	*6 (0.8)*		*0 (0)*	*0 (0)*		
Surgical procedure							
RAMIE	47 (78.3)	73 (9.2)	**0.001**	46 (78.0)	16 (9.0)	**0.001**	–
MIE	13 (21.7)	709 (89.1)		13 (22.0)	161 (91.0)		
Hybrid	0 (0.0)	14 (1.7)		0 (0.0)			
Location anastomosis							
Cervical	12 (20.0)	180 (22.8)	**0.002**	12 (20.3)	39 (22.0)	0.784	0.04
Intrathoracic	47 (78.3)	616 (77.3)		47 (79.7)	138 (78.0)		
No anastomosis	1 (1.7)	0 (0)		–	–		

Data are presented as *n* (%) or mean ± standard deviation unless otherwise indicated. Bold *p*-values are statistically significant

ASA, American Society of Anesthesiologists; BMI, body mass index; CI, confidence interval; GEJ, gastro esophageal junction; ICG, indocyanine green; MIE, minimally invasive esophagectomy; RAMIE, robot assisted minimally invasive esophagectomy; SCC, squamous cell carcinoma; WHO, World Health Organization

## Results

### Study Population

**Fig. 1:**
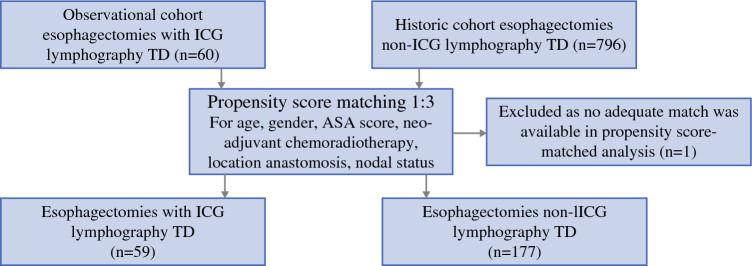
Flowchart of patient selection

Between May 2022 and August 2023, a total of 60 patients underwent ICG fluorescence lymphography. After propensity score matching, 59 of these patients were matched to a historical cohort of 177 patients who did not undergo ICG lymphography. One of 60 patients could not be matched. Adequate balance was achieved for all baseline characteristics after propensity score matching (Fig. 1).

Mean age was 67.0 (standard deviation [SD] 8.4) in the ICG group versus 66.7 years in the non-ICG group (SD 9.1) (*p* = 0.827), and mean body mass index was 25.8 kg/m^2^ (SD 4.7) versus 24.8 kg/m^2^ (SD 3.7) (*p* = 0.118). Tumors were located distally in 86.4% of the ICG group versus 71.2% in the non-ICG group (*p* = 0.007), and histopathology was adenocarcinoma in 93.2% versus 80.2%, respectively (*p* = 0.058). Chemoradiotherapy was administered to 93.2% versus 93.8% (*p* = 0.878). The surgical procedure differed significantly, with robotic-assisted minimally invasive esophagectomy performed in 78.0% of ICG patients and 9.0% of non-ICG patients (*p* = 0.001) (Table 1).

### ICG Lymphography

ICG was administered in the inguinal lymph nodes (26 patients, 44.1%), small bowel mesentery (30 patients, 50.8%) or both (three patients, 5.1%) (Table 2). Thoracic duct visualization was successful in 50 of 59 patients (84.7%), borderline in four patients (6.8%), and absent in five patients (8.5%) (all absent signal was after solely inguinal administration). Fluorescence imaging influenced operative management in 21 patients (35.6%) (Table 2). In two cases, leakage was identified at the clip closure site, requiring additional clip(s). In one case, a double drainage system was identified and also clipped. In the remaining 18 cases, an extra metal clip or a hemolock was applied to a collateral to reinforce lymphatic sealing and prevent postoperative leakage. The median duration of thoracic drainage of patients in the ICG group was 6 days (range 1–17). No ICG-related adverse events were reported.

**Table 2:** Characteristics of indocyanine green (ICG) administration and chyle leakage

Characteristic	ICG group (*N* = 59)
*Administration site*	
Inguinal lymph nodes	26 (44.1)
Mesentery	33 (55.9)
*Thoracic duct visualization quality*	
Clear	50 (84.7)
Borderline	4 (6.8)
None	5 (8.5)
Change of management	21 (35.6)
Duration of thoracic drainage, days	6 (1–17)
Adverse events to ICG	0 (0)

Data are presented as *n* (%) or median (range)

### Postoperative Outcomes

Chyle leakage occurred in 10 patients (16.9%) in the ICG group versus 18 patients (10.2%) in the non-ICG group (*p* = 0.163) (Table 3). Chyle leakage was classified as type I in seven (70.0%) affected patients in the ICG group and 15 (83.3%) in the non-ICG group (*p* = 0.410) and type II in three (30.0%) of the ICG group and one (5.6%) of the non-ICG group (*p* = 0.077). Type III was not seen in the ICG group and reported twice in the non-ICG group (11.1%, *p* = 0.271). Of those, one chyle leak required radiological drainage, and the other necessitated surgical reintervention.

**Table 3:** Postoperative outcomes after propensity score matching

Outcome	ICG group ( *N* = 59)	Non-ICG group ( *N* = 177)	*p*-value
Postoperative complication	39 (66.1)	66 (62.7)	0.639
Pneumonia	5 (8.5)	33 (18.6)	0.066
Anastomotic leakage	6 (10.2)	30 (16.9)	0.210
Recurrent nerve palsy	5 (8.5)	7 (4.0)	0.171
Chyle leakage	10 (16.9)	18 (10.2)	0.163
ECCG type I	7 (70.0)	15 (83.3)	0.410
A^a^	4 (40.0)	12 (66.7)	
B^b^	3 (30.0)	3 (16.7)	
ECCG type II	3 (30.0)	1 (5.6)	0.077
A^a^	2 (20.0)	1 (5.6)	
B^b^	1 (10.0)	0 (0.0)	
ECCG type III	0 (0)	2 (11.1)	0.271
A^a^	0 (0)	1 (5.6)	
B^b^	0 (0)	1 (5.6)	
Duration of thoracic drainage	6.0 (1–17)	5.0 (1–28)	**<0.001**
Duration of hospital stay, days	8.0 (5–83)	10.0 (3–109)	**0.023**
Readmission rate	10 (17.5)	30 (17.6)	0.712
Readmission due to chyle leak	0 (0.0)	1 (3.3)	0.601
(y)pT-stage			0.460
T0	13 (22.0)	40 (23.8)	
T1	12 (20.3)	35 (20.8)	
T2	15 (25.4)	25 (14.9)	
T3	18 (30.5)	65 (38.7)	
T4	1 (1.7)	3 (1.8)	
(y)pN-stage			0.435
T0	37 (62.7)	104 (61.2)	
T1	7 (11.9)	32 (18.8)	
T2	12 (20.3)	23 (13.5)	
T3	3 (5.1)	11 (6.5)	
Lymph node yield	35.0 (18–64)	34.5 (7–73)	0.210
Positive lymph node yield	0 (0–16)	0 (0–31)	0.983
Radicality (R0)	57 (96.6)	171 (96.6)	1.000

Data are presented as *n* (%) or median (range) unless otherwise indicated. Bold *p*-values are statistically significant

ECCG, Esophagectomy Complications Consensus Group; (y)*p*, (post-neoadjuvant) pathological

^a^Output chyle leakage <1 L/day

^b^Output chyle leakage >1 L/day

Among the nine patients in whom fluorescence visualization was inadequate or absent, two ultimately developed postoperative chyle leakage. Interestingly, the majority of chyle leakage cases (eight patients) occurred despite successful identification of the thoracic duct using fluorescence guidance. This suggests that, although fluorescence imaging can aid in the intraoperative localization of the thoracic duct, the absence of a clear fluorescent signal does not necessarily predict the occurrence of chyle leakage.

Within patients in the ICG group, chyle leakage was observed in three patients (14.3%) among those who had extra clip placement/changed management and in seven (18.4%) of those who did not have changed management (*p* = 0.685) (Table 4). The median duration of thoracic drainage was 6.0 days (range 1–17) in the ICG group and 5.0 days (range 1–28) in the non-ICG group (*p*<0.001). The median duration of hospital stay was 8.0 days (range 5–83) in the ICG group and 10.0 days (range 3–109) in the non-ICG group (*p* = 0.023). There were no differences in readmission rates, (positive) median lymph node yield, and radicality (R0 resection) (Table 3).

**Table 4:** Outcomes after change of management due to indocyanine green (ICG) per operatively

ICG group (*N* = 59)	Changed management ( *N* = 21)	Unchanged management ( *N* = 38)	*p*-value
Chyle leakage			0.685
Yes	3 (14.3)	7 (18.4)	
No	31 (81.6)	18 (85.7)	

Data are presented as *n* (%) unless otherwise indicated.

## Discussion

This large observational propensity score matched cohort study assessed the efficacy of intraoperative fluorescence lymphography with ICG in preventing chyle leakage after minimally invasive esophagectomy. Although it did not reduce the incidence of chyle leakage, it confirmed the technique’s safety and real-time utility. Fluorescence lymphography allowed for accurate visualization of the thoracic duct, its collaterals, and potential leakage sites and regularly led to a change in operative management.

Two ICG administration techniques were used: bilateral injection in the inguinal lymph nodes and injection into small bowel mesentery. The rate of successful fluorescence thoracic duct identification was 85%, which is at the lower end of the spectrum reported in the literature. Reported rates vary between 70% and 100%, depending on factors such as injection site, timing, dosage, and dilution.^14,20,21^

The incidence of postoperative chyle leakage was not reduced in the ICG cohort. This contrasts with the finding of Vecchiato et al.^14^, whose cohort reported a 0% incidence of chyle leakage after using fluorescence. Puccetti et al.^22^ demonstrated a significant reduction in the incidence of chyle leakage from 11.8% in the non-ICG group to 4.6% in the ICG group (*p*=0.026). Fluorescence lymphography did influence intraoperative management, potentially mitigating leakage risk in patients with initially worse prognostic factors. The ability of ICG to equalize the risk of chyle leakage across patients with changed management during surgery versus no change in management underscores its clinical relevance despite the lack of a significant overall reduction in leakage incidence.

The absence of a significant decrease in postoperative chyle leakage in the ICG group may at first be attributed to a skewed distribution of robot-assisted minimally invasive esophagectomies across the groups. The ICG group consisted of more robotic-assisted minimally invasive esophagectomies, a technically challenging technique that is inextricably linked to a learning curve, which could have masked the potential effect of ICG in reducing chyle leakage.^23^ Although some degree of heterogeneity in surgical approach (minimally invasive vs. robotic-assisted, location of anastomosis) and ICG administration existed, all procedures followed a standardized esophagectomy with lymphadenectomy with standard en-bloc thoracic duct resection protocol. Besides, propensity score matching was applied to minimize baseline differences, enabling a robust comparison of outcomes. Furthermore, the prospective nature of the study may have provided more alert and precise monitoring and documentation of chyle leakage compared with the historical cohort. Additionally, the extensive lymphadenectomy performed in our center could contribute to chyle leakage from alternative lymphatic pathways. The ICG group included a higher proportion of distal tumors and adenocarcinomas. Although these were not necessarily associated with higher staged or more advanced tumors, the more extensive lymphadenectomy in the abdomen and thorax in more recent patients could result in higher chyle leakage rates in the ICG cohort. Also, excessive clip placement may have resulted in localized obstruction, increasing upstream lymphatic pressure, potentially causing leakage at other sites, although we did not measure the number of clips used during non-ICG thoracic duct resection. The variability in handled definitions of chyle leakage across studies complicates comparisons and leads to a wide range of incidence figures across the literature. The current study adhered to robust and precise global ECCG definitions, which may contribute to our relatively high reported rates, raising questions about the comparability of these findings with those of other studies.^24^ All these factors collectively underscore the complexity of the issue and highlight the need for further research and standardization of outcome measures. Despite the higher incidence of postoperative chyle leakage observed in the ICG cohort, all cases were successfully managed conservatively, unlike all non-ICG cases, avoiding the need for radiological or surgical re-interventions. Future studies should focus on establishing evidence-based and standardized ICG administration protocols to maximize its utility in intraoperative lymphography.

The intraoperative insights provided by ICG fluorescence lymphography may have contributed to improved postoperative care, as reflected in less severe chyle leaks and a modest 2-day reduction in median hospital stay in the ICG group. Nevertheless, this observation requires cautious interpretation. The ICG cohort represents a more recent patient population, which introduces a potential confounding factor. Improvements in perioperative management over time, particularly the progressive adoption and refinement of Enhanced Recovery After Surgery protocols may have independently contributed to shortened hospital stays. This 2-day reduced hospital stay has clear implications for patient recovery, quality of life, and healthcare resource utilization and might highlight that fluorescence lymphography could be a cost-effective adjunct in esophageal surgery. However, the duration of thoracic drainage was not reduced, leaving uncertainty as to whether the shorter hospitalization can be directly attributed to ICG lymphography.

This study has several limitations. First, despite being a prospective cohort study with propensity score matching, it remains non-randomized and is therefore subject to the inherent biases associated with retrospective matching. Residual confounding may persist because of differences in prospective and retrospective outcome registration and perioperative management changes over time. Additionally, in the retrospective cohort, chyle leakage and drainage characteristics may have been incompletely documented, as some patient records were paper based. This could have introduced information bias, potentially underestimating the true incidence of chyle leakage in the non-ICG cohort. Moreover, chyle leakage after esophagectomy is influenced by thoracic duct management, as reflected in a recent systematic review showing a higher incidence after thoracic duct resection than with preservation.^25^ Future studies will evaluate whether fluorescence-guided lymphography has a different value within distinct thoracic duct management strategies. Therefore, a randomized study is designed to help minimize this limitation. Fourth, the routine use of Jackson–Pratt drains only became standard practice in our center from 2021 onwards. This shift may have influenced postoperative outcomes, particularly in diagnosing chyle leaks and length of hospital stay. Moreover, logistical constraints prevented the formation of a strictly consecutive cohort, leading to some temporal overlap between groups. However, this overlap may have partially mitigated selection bias.

In conclusion, this study confirms that real-time ICG fluorescence lymphography is a safe and effective technique for accurate visualization of the thoracic duct, guiding its resection. However, a significant reduction in postoperative chyle leakage could not be demonstrated, questioning its broader clinical significance. The observed reduced hospital stay in the ICG group could have been a result of less severe leakages and therewith less severe clinical courses, facilitating faster recovery. However, as the ICG group represents a more recent cohort, perioperative management changes over time that might have contributed to the shorter hospital stay could not be excluded. Further investigation, including the upcoming TUPEC trial, which randomizes thoracic duct resection versus preservation with ICG lymphography used in both arms, is essential to determine its true efficacy in reducing chyle leakage and to better define its role in esophageal cancer surgery. The trial’s robust design may help clarify the value of thoracic duct resection and the potential contribution of ICG lymphography, thereby providing a solid framework for evidence-based recommendations.

## Supplementary Information

Below is the link to the electronic supplementary material.

Supplementary file1 (DOCX 78 KB)

## References

[1] Defize IL, Gorgels SMC, Mazza E, Schurink B, Strignano P, Catalano G, et al. The presence of metastatic thoracic duct lymph nodes in western esophageal cancer patients: a multinational observational study. *Ann Thorac Surg*. 2022;113(2):429–35. 33676903 10.1016/j.athoracsur.2021.02.041

[2] Schurink B, Defize IL, Mazza E, Ruurda JP, Brosens LAA, Roeling TAP, et al. Two-field lymphadenectomy during esophagectomy: the presence of thoracic duct lymph nodes. *Ann Thorac Surg*. 2018;106(2):435–9. 29580778 10.1016/j.athoracsur.2018.02.047

[3] Ilahi M, St Lucia K, Ilahi TB. Anatomy, thorax, thoracic duct. Treasure Island: StatPearls; 2024. 30020599

[4] Defize IL, Schurink B, Weijs TJ, Roeling TAP, Ruurda JP, van Hillegersberg R, et al. The anatomy of the thoracic duct at the level of the diaphragm: a cadaver study. *Ann Anat*. 2018;217:47–53. 29510243 10.1016/j.aanat.2018.02.003

[5] Schafrat PJM, Henckens SPG, Hagens ERC, Eshuis WJ, Gisbertz SS, Lameris W, et al. Clinical implications of chyle leakage following esophagectomy. *Dis Esophagus*. 2023;36(2):doac047. 35830862 10.1093/dote/doac047 PMC9885733

[6] Kranzfelder M, Gertler R, Hapfelmeier A, Friess H, Feith M. Chylothorax after esophagectomy for cancer: impact of the surgical approach and neoadjuvant treatment: systematic review and institutional analysis. *Surg Endosc*. 2013;27(10):3530–8. 23708712 10.1007/s00464-013-2991-7

[7] Low DE, Kuppusamy MK, Alderson D, Cecconello I, Chang AC, Darling G, et al. Benchmarking complications associated with esophagectomy. *Ann Surg*. 2019;269(2):291–8. 29206677 10.1097/SLA.0000000000002611

[8] Nederlof N, Slaman AE, van Hagen P, van der Gaast A, Slankamenac K, Gisbertz SS, et al. Using the comprehensive complication index to assess the impact of neoadjuvant chemoradiotherapy on complication severity after esophagectomy for cancer. *Ann Surg Oncol*. 2016;23(12):3964–71. 27301849 10.1245/s10434-016-5291-3 PMC5047940

[9] Weijs TJ, Ruurda JP, Broekhuizen ME, Bracco Gartner TCL, van Hillegersberg R. Outcome of a step-up treatment strategy for chyle leakage after esophagectomy. *Ann Thorac Surg*. 2017;104(2):477–84. 28499656 10.1016/j.athoracsur.2017.01.117

[10] Hagens ERC, Feenstra ML, Eshuis WJ, Hulshof M, van Laarhoven HWM, van Berge Henegouwen MI, et al. Conditional survival after neoadjuvant chemoradiotherapy and surgery for oesophageal cancer. *Br J Surg*. 2020;107(8):1053–61. 32017047 10.1002/bjs.11476 PMC7317937

[11] Lagarde SM, Omloo JM, de Jong K, Busch OR, Obertop H, van Lanschot JJ. Incidence and management of chyle leakage after esophagectomy. *Ann Thorac Surg*. 2005;80(2):449–54. 16039184 10.1016/j.athoracsur.2005.02.076

[12] Kamarajah SK, Siddaiah-Subramanya M, Parente A, Evans RPT, Adeyeye A, Ainsworth A, et al. Risk factors, diagnosis and management of chyle leak following esophagectomy for cancers: an international consensus statement. *Ann Surg Open*. 2022;3(3):e192. 36199483 10.1097/AS9.0000000000000192 PMC9508983

[13] Yang F, Zhou J, Li H, Yang F, Xiao R, Chi C, et al. Near-infrared fluorescence-guided thoracoscopic surgical intervention for postoperative chylothorax. *Interact Cardiovasc Thorac Surg*. 2018;26(2):171–5. 29049798 10.1093/icvts/ivx304

[14] Vecchiato M, Martino A, Sponza M, Uzzau A, Ziccarelli A, Marchesi F, et al. Thoracic duct identification with indocyanine green fluorescence during minimally invasive esophagectomy with patient in prone position. *Dis Esophagus*. 2020;33(12):doaa030. 32448899 10.1093/dote/doaa030 PMC7720005

[15] Ghaferi AA, Schwartz TA, Pawlik TM. STROBE reporting guidelines for observational studies. *JAMA Surg*. 2021;156(6):577–8. 33825815 10.1001/jamasurg.2021.0528

[16] Hagens ERC, van Berge Henegouwen MI, van Sandick JW, Cuesta MA, van der Peet DL, Heisterkamp J, et al. Distribution of lymph node metastases in esophageal carcinoma [TIGER study]: study protocol of a multinational observational study. *BMC Cancer*. 2019;19(1):662. 31272485 10.1186/s12885-019-5761-7 PMC6610993

[17] Henckens SPG, Hagens ERC, van Berge Henegouwen MI, Meijer SL, Eshuis WJ, Gisbertz SS. Impact of increasing lymph node yield on staging, morbidity and survival after esophagectomy for esophageal adenocarcinoma. *Eur J Surg Oncol*. 2023;49(1):89–96. 35933270 10.1016/j.ejso.2022.07.012

[18] Cichero JA, Lam P, Steele CM, Hanson B, Chen J, Dantas RO, et al. Development of international terminology and definitions for texture-modified foods and thickened fluids used in dysphagia management: the IDDSI framework. *Dysphagia*. 2017;32(2):293–314. 27913916 10.1007/s00455-016-9758-y PMC5380696

[19] Low DE, Alderson D, Cecconello I, Chang AC, Darling GE, D’Journo XB, et al. International consensus on standardization of data collection for complications associated with esophagectomy: esophagectomy complications consensus group (ECCG). *Ann Surg*. 2015;262(2):286–94. 25607756 10.1097/SLA.0000000000001098

[20] Tokumaru S, Kitazawa M, Nakamura S, Koyama M, Soejima Y. Intraoperative visualization of morphological patterns of the thoracic duct by subcutaneous inguinal injection of indocyanine green in esophagectomy for esophageal cancer. *Ann Gastroenterol Surg*. 2022;6(6):873–9. 36338584 10.1002/ags3.12594 PMC9628221

[21] Barnes TG, MacGregor T, Sgromo B, Maynard ND, Gillies RS. Near infra-red fluorescence identification of the thoracic duct to prevent chyle leaks during oesophagectomy. *Surg Endosc*. 2022;36(7):5319–25. 34905086 10.1007/s00464-021-08912-1 PMC9160097

[22] Puccetti F, Cinelli L, Barbieri LA, Socci D, Clelia DS, De Cobelli F, et al. The near-infrared visualization and preemptive ligation of the thoracic duct effectively reduce the chyle leak incidence after minimally invasive esophagectomy. *Ann Surg*. 2024;280(5):780–7. 39140608 10.1097/SLA.0000000000006490

[23] Pickering OJ, van Boxel GI, Carter NC, Mercer SJ, Knight BC, Pucher PH. Learning curve for adoption of robot-assisted minimally invasive esophagectomy: a systematic review of oncological, clinical, and efficiency outcomes. *Dis Esophagus*. 2023;36(6):doac089. 36572404 10.1093/dote/doac089

[24] Power R, Smyth P, Donlon NE, Nugent T, Donohoe CL, Reynolds JV. Management of chyle leaks following esophageal resection: a systematic review. *Dis Esophagus*. 2021;34(11):doab012. 33723611 10.1093/dote/doab012 PMC8597908

[25] Nijssen DJ, van der Aa DC, Ali M, Kazemier G, Jamaludin FS, Eshuis WJ, et al. Resection vs. ligation vs. preservation of the thoracic duct during esophagectomy for cancer: a systematic review and meta-analysis. *Cancers (Basel)*. 2025;17(6):967. 40149302 10.3390/cancers17060967 PMC11940447

