# Supplementary figures and images for: Fluorescence Lymphography Using Indocyanine Green During Esophagectomy for Cancer to Prevent Chyle Leakage: A Propensity Score Matched Analysis
Source: Ann Surg Oncol. 2026 Mar 19;33(7):6233–41. doi: 10.1245/s10434-026-19359-w (PMC13242416; doi:10.1245/s10434-026-19359-w)

| 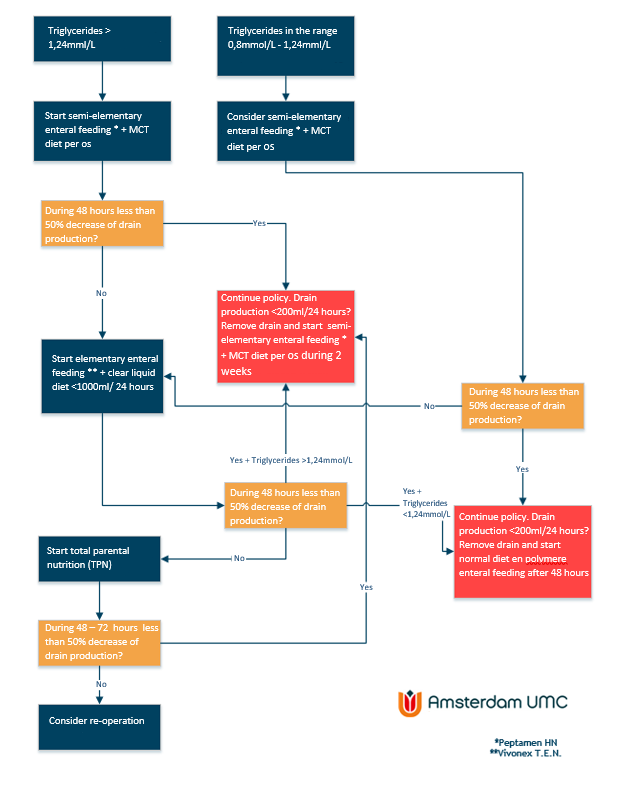 |
| --- |
| **Supplementary Figure 1.** Step-by-step plan of treatment chyle leakage within Amsterdam UMC |

Supplement: Supplementary file 1 — Supplementary file1 (DOCX 78 KB) [file 10434_2026_19359_MOESM1_ESM.docx]
